# The Hospitals Association

**Published:** 1901-11-02

**Authors:** 


### THE HOSPITALS ASSOCIATION.

The sixteenth annual meeting of the Hospitals Association was held at the Hospital Buildings, Southampton Street, the chair being taken by Sir Savile Crossley, M.P., the President.

The Report presented stated that the council had not deemed it necessary to call a general meeting of the members during the year, there having been no matters of immediate public interest demanding discussion. Regret was expressed that in consequence of the pressure of public business and the failure of the Government to give any special facilities to the Bill introduced by the General Hospital Council for London for relieving hospitals from local rates was not passed or discussed. It was hoped, however, that further efforts would be made next session. The report went on to remind members that some years ago the Association received through the instrumentality of the Deputy-Chairman of the Council (Sir Henry Burdett) a gift of £600 in trust to establish a reference library on all subjects connected with hospitals and charities generally. Pending the drawing up of a complete scheme the money was allowed to accumulate. Meanwhile the nucleus of a library was obtained by the deputy-chairman presenting to the Association a valuable and extensive series of hospital reports and rules for all parts of the world which he had had bound at his own expense; and early this year the Council decided to spend £120 out of the accumulated funds in the purchase of standard works on hospital administration, construction, finance, statistics, and nursing. A special committee was appointed to manage the library, and it was proposed to add from time to time such books as might be published or might be suggested by the members should they be considered of sufficient general interest. By arrangement with Sir Henry Burdett the library would be worked in conjunction with the Hospital Library and Charities Bureau, which would appoint and pay the librarian charged with the custody of the books. The council had now great satisfaction in reporting that the library was opened on October 1st ult. The want of such a centre of reference had long been felt, and the council felt sure it would be of great service to all connected with and interested in hospitals and charitable institutions.

The report of the committee of the Street Ambulance Branch stated that the operations of the London Street Ambulance Service during the year had exceeded those of either of the nine years preceding, during which the branch had been at work, each year's returns showing an increase in the number of times the ambulances had been used. There are 51 stations in the Metropolis and 2,404 cases were attended to in 1900–1, the total number since the establishment of the service in 1891 having been 17,775. These figures are approximate only. At fire brigade stations a careful record is kept of the use of the ambulance; the City police also do so as well as some of the hospitals, but for the most part estimates only were possible.

The adoption of these reports was briefly moved by the CHAIRMAN, and in supporting the motion, Sir HENRY BURDETT reminded the meeting that it had long been their wish that there should be a centre of reference to which any one who wanted accurate information on a particular subject connected with hospital work could apply. The opening of the Charities Bureau marked an important departure, recognising the fact that now all workers desired efficiency in administration and construction and were willing to co-operate. The gentleman appointed to take charge of the library and the bureau had long been connected with hospital work, and as he would be there every day he would be able to render considerable assistance to inquirers. Sir Henry expressed the belief that before long those interested in hospitals would wonder how they got along without such a centre. In the matter of building construction especially, he thought it would be very useful and should have an important effect on the economy and efficiency of hospital buildings. As to the Ambulance Branch, something like one-half of all the street accidents in London were handled by it; the numbers of cases showed an increase of 150 per cent. since the work was commenced, and in fact the service was so efficient that practically it did deal with all the accidents in the streets of London. Before its establishment there was no other way to deal with them than removal in a cab to a hospital, and it was frequently found that owing to the jolting and awkwardness of the vehicle simple fractures became compound and even complicated—often with fatal results. London owed it to Mr. H. L. Bischoffsheim, the treasurer, who defrayed all the expenses, and to Mr. Thomas Ryan, the hon. secretary, who had voluntarily undertaken the organisation of the 51 stations all over the metropolis. He might mention that Mr. Bischoffsheim was also prepared to find the whole of the money required to equip horse ambulances if it could be shown that they were necessary or desirable. So far, however, the hand service had been found to be adequate and more adaptable.

The reports were adopted, after which Sir HENRY BURDETT moved the re-election of Sir Savile Crossley as President of the Association. He was chairman of the Hospital Saturday Fund, a member of the council of the Metropolitan Hospital Sunday Fund, hon. sec. of the Prince of Wales's Hospital Fund for London, and one of the most useful members of their council. The mere recital of these offices showed the estimation he was held in in the hospital world, and it was therefore fitting that he should be president of that Association which united all hospital workers under its ægis. And he was the gentleman who first commenced to give large sums annually through the Sunday Fund—an example which had been followed by others and would, he hoped, be still more largely followed.

Surgeon-Colonel INCE seconded the motion which was carried.

Mr. MICHELLI proposed the re-election of the officers and said he looked forward to the time when the Association would move again with renewed vigour. He recalled the steps taken by it in the past, resulting in the passing of the Mortmain and Charitable Uses Act. Much cold water was thrown on those efforts, but they succeeded, and he had no doubt they would also succeed in the matter they now had in hand—the exemption of hospitals from local rating, as to which the public were in a state of great ignorance.

The motion was carried, and the proceedings terminated with a cordial vote of thanks to the Chairman.

